# Efficacy of Epimedii Folium and related preparations in experimental osteoporosis: a systematic review and meta-analysis

**DOI:** 10.3389/fphar.2026.1951582

**Published:** 2026-10-14

**Authors:** Zuowen Wang, Ningchuan Zhang, Junxiao Ren, Chun Li

**Affiliations:** 1 Department of Orthopedics, Ziyang Hospital of Sichuan Provincial People’s Hospital Medical Group, Ziyang, Sichuan, China; 2 The Clinical Medical College, Chengdu University of Traditional Chinese Medicine, Chengdu, Sichuan, China

**Keywords:** bone mineral density, Epimedii Folium, icariin, meta-analysis, osteoporosis

## Abstract

**Background:**

*Epimedii Folium* and related extracts have shown osteogenic, antiresorptive, antioxidant, and anti-inflammatory activities in experimental studies. This systematic review and meta-analysis evaluated their efficacy in rodent models of osteoporosis.

**Methods:**

PubMed, Embase, Web of Science, China National Knowledge Infrastructure, and Wanfang Data were searched from their inception to 13 June 2026. Controlled *in vivo* studies using rat or mouse models of osteoporosis or experimentally induced bone loss were eligible. Random-effects models generated Hedges’ standardized mean differences (SMDs) with 95% confidence intervals (CIs). The risk of bias was assessed using the SYRCLE tool. The protocol was registered in PROSPERO (CRD420261428223). Sensitivity analyses examined heterogeneity, the influence of the Qi 2019 estimate, and the effects of multi-arm dependence, preparation type, disease model, duration, and dose. Certainty was appraised using an adaptation of GRADE for preclinical animal evidence.

**Results:**

Twenty-nine independent rodent studies were included. Femoral BMD increased (20 comparisons from 9 studies; SMD = 1.66, 95% CI: 1.07–2.25; 95% prediction interval [PI]: −0.61 to 3.93; I^2^ = 73.77%). Excluding Qi 2019 reduced the SMD to 1.44 (95% CI: 0.96–1.92), while a three-level study-cluster robust model yielded an SMD of 2.25 (95% CI: 0.40–4.10). The average effects favored several regional BMD, microarchitectural, and biomechanical outcomes, but the majority of PIs crossed the null. PINP was not significant. The Systematic Review Centre for Laboratory Animal Experimentation (SYRCLE) assessment found that the majority of judgments were unclear, with nine domain-level judgments rated as high risk; certainty was very low.

**Conclusion:**

Random-effects pooled estimates favored the examined oral Epimedii Folium-derived preparations for several regional BMD, trabecular microarchitecture, and *ex vivo* biomechanical outcomes in rodent models. However, wide prediction intervals, chemical and model heterogeneity, small study sizes, incomplete reporting, and correlated multi-arm comparisons limit confidence in the pooled effect magnitudes. Chemically standardized preparations and exposure-informed dosing are needed before clinical translation.

**Systematic Review Registration:**

https://www.crd.york.ac.uk/PROSPERO/view/CRD420261428223, identifier CRD420261428223.

## 1 Introduction

Osteoporosis is a systemic skeletal disorder in which reduced bone mass, cortical and trabecular deterioration, and impaired material properties combine to increase the risk of fragility fractures. Its clinical importance is not captured by BMD alone: vertebral and hip fractures lead to pain, deformity, loss of independence, excess mortality, and high long-term healthcare use. Population aging, the persistent treatment gap after fragility fracture, and poor adherence to long-term therapy mean that the absolute burden continues to rise despite the availability of effective antiresorptive and anabolic agents (Compston et al., 2019; Johnston and Dagar, 2020; LeBoff et al., 2022; Ebeling et al., 2022).

Bone remodeling is governed by coupled communication among osteoclasts, osteoblasts, osteocytes, marrow stromal cells, immune cells, and vascular cells (Kim et al., 2020). RANKL-driven osteoclastogenesis, OPG-mediated restraint, canonical Wnt/beta-catenin signaling, BMP/Smad/Runx2 transcription, PI3K/AKT-dependent survival, and redox-sensitive pathways jointly determine whether each remodeling cycle produces a net gain or loss (Boyce and Xing, 2008). Estrogen deficiency amplifies RANKL and inflammatory cytokines, increases oxidative stress, prolongs osteoclast survival, and impairs osteoblast and osteocyte function (Almeida et al., 2017; Manolagas et al., 2013; Weitzmann and Pacifici, 2006). Glucocorticoids, diabetes, immobilization, aging, chronic inflammation, and nutritional or metabolic disturbances create partially distinct but overlapping mechanisms of secondary bone loss (Ebeling et al., 2022; Bandeira et al., 2022; Kverka and Stepan, 2024; Polyzos et al., 2021).

Current treatments substantially reduce fracture risk when selected appropriately, but no single therapy is ideal for every patient (LeBoff et al., 2022; Eastell et al., 2019). Bisphosphonates may be limited by gastrointestinal intolerance and rare skeletal adverse events; denosumab requires carefully planned sequential therapy to prevent rebound bone turnover; anabolic therapies are costly and time-limited; and real-world adherence remains suboptimal (Anastasilakis et al., 2017). These limitations do not diminish the value of standard therapy but provide a rationale for investigating chemically defined natural products as pharmacological leads or adjunctive strategies, provided that their efficacy, exposure, composition, and safety are evaluated with the same rigor expected for conventional agents.

*Epimedii Folium*, commonly known as “Yin yang huo,” is derived from medicinal *Epimedium* species and has a long ethnomedical history in East Asia for treating conditions described as weakness of the bones and tendons, soreness of the lumbar region and knees, and impaired mobility (Cui, 2024; I et al., 2016). Modern phytochemical research has identified prenylated flavonol glycosides as characteristic constituents. Icariin is among the most extensively studied compounds and is widely used as a quality-control marker, while icariside II, icaritin, epimedin A–C, and related metabolites contribute to the pharmacological profile. The plant’s traditional use and phytochemical composition provide a direct ethnopharmacological rationale for evaluating its activity related to bone health (Cui, 2024; Wang et al., 2018; Zhang et al., 2023a; Hu et al., 2025). Traditional forms include decoctions and processed leaves. The Chinese Pharmacopoeia recognizes *Epimedium brevicornu* Maxim., *Epimedium sagittatum* (Siebold & Zucc.) Maxim., *Epimedium pubescens* Maxim., and *Epimedium koreanum* Nakai (Berberidaceae) as origins of *Epimedii Folium*; *Epimedium wushanense* T.S. Ying is designated for *Epimedii Folium Wushanensis*. Processing with refined mutton fat can alter these plants’ chemical and biopharmaceutical properties (Zhang et al., 2022; He et al., 2020).

Icariin has been reported to promote osteoblast lineage commitment and matrix mineralization via BMP-2/Smad/Runx2, Wnt/beta-catenin, PI3K/AKT, alpha-enolase, microRNA, circRNA, SIRT1, and BMAL1-dependent mechanisms (Ai et al., 2023; Cao et al., 2012; Huang et al., 2020). It may also reduce osteoclast differentiation by modulating OPG/RANKL, NF-kappaB, MAPK, c-Fos/NFATc1, and cathepsin K-related pathways (Boyce and Xing, 2008; Bi et al., 2022; Hu et al., 2007; Wang et al., 2020). Its antioxidant and anti-inflammatory effects, including Nrf2 activation, ferroptosis suppression, and reduced inflammatory cytokine signaling, may preserve osteoblast and BMSC viability under estrogen-deficient, diabetic, aging, or disuse conditions (Wang et al., 2020).

Translation is nevertheless complicated by low aqueous solubility, extensive intestinal deglycosylation, species-dependent metabolism, and the variable composition of crude and enriched *Epimedium* preparations (Gao and Zhang, 2022; Li et al., 2015; Szabo et al., 2022). Administered mass is therefore an imperfect surrogate for systemic exposure, especially when purified icariin is pooled with total flavonoid fractions or crude extracts (Meng et al., 2021; Teo et al., 2019). Human pharmacokinetic studies of standardized *Epimedium* preparations and experimental delivery platforms illustrate both the potential and the uncertainty of exposure optimization (Ding et al., 2023).

The present review therefore aimed to broaden the synthesis of preclinical evidence. The primary objective was to quantify the effects on site-specific BMD and trabecular microarchitecture. The secondary objectives were to examine biomechanical properties and bone turnover markers, characterize the methodological risk of bias, assess robustness and small-study effects, and interpret the pooled findings in relation to the current mechanistic, pharmacokinetic, and translational evidence. The most direct prior benchmark was a 2016 meta-analysis of seven studies of icariin in ovariectomized rats (Xu et al., 2016). The present review extends that work by including 29 studies across multiple preparations and bone-loss models, separating anatomical and structural outcomes, and examining botanical identity, dependence, prediction intervals, moderators, and certainty.

## 2 Methods

### 2.1 Review design and search strategy

This systematic review was prepared in accordance with the PRISMA 2020 statement (Page et al., 2021) and the guidance for evidence synthesis in animal studies. The protocol was registered in the International Prospective Register of Systematic Reviews (PROSPERO; CRD420261428223). The PICOS review question was defined as follows: rats or mice with experimental osteoporosis or pathological bone loss (population); orally administered Epimedii Folium, *Epimedium* extracts, or flavonoid fractions, or icariin (intervention); untreated or vehicle-treated disease-model controls (comparator); skeletal and bone-metabolism outcomes (outcomes); and controlled *in vivo* studies (study design). PubMed, Web of Science, Embase, China National Knowledge Infrastructure (CNKI), and Wanfang Data were searched from their inception to 13 June 2026, with no language restrictions. Reference lists of eligible reports and relevant reviews were screened for additional studies. The search concepts and working queries are provided in Supplementary Material S1.

### 2.2 Eligibility criteria

Studies were eligible when they (Compston et al., 2019) used an *in vivo* rat or mouse model of osteoporosis or experimentally induced pathological bone loss (Johnston and Dagar, 2020); included a disease-model control group (LeBoff et al., 2022); evaluated a single-herb *Epimedium* preparation, a separable *Epimedium* extract, or flavonoid fraction, or icariin administered by an enteral route (oral administration, gavage, or diet); and (Ebeling et al., 2022) reported at least one quantitative outcome related to BMD, trabecular microarchitecture, histomorphometry, bone biomechanics, or bone metabolism. Ovariectomy, retinoic acid exposure, iron overload, diabetes, hindlimb suspension, thioacetamide exposure, and genetic OPG deficiency were accepted because each produced a defined bone-loss phenotype. *In vitro*-only studies, clinical studies, reviews, duplicated reports, positive-control arms, and inseparable multi-herb combinations were excluded from the quantitative synthesis. When a study contained both eligible and ineligible intervention arms, only the eligible *Epimedium*-derived arm was extracted.

### 2.3 Study selection and data extraction

Two reviewers independently screened titles and abstracts, assessed full texts, and extracted study characteristics and outcomes into a standardized workbook. The variables included the Study ID, species, model, intervention and preparation, dose, duration, group size, anatomical site, outcome definition, mean, and dispersion. Disagreements were resolved by consensus and, when necessary, through consultation with a third reviewer. Values reported in text or tables were transcribed directly. When only graphs were available, WebPlotDigitizer 4.7 was used to digitize bar heights and error bars. Standard errors were converted to standard deviations using SD = SEM × √n. Missing group sizes, unclear error types, and irrecoverable values were not imputed. Each anatomical site and outcome was retained as a distinct effect-size record. Of the 374 extracted comparisons, 258 (69.0%) came from text or tables, and 116 (31.0%) came from graphs. All pooled graph-derived effect sizes with |g| > 5 were rechecked against the source figure, caption, error bar definition, and sample size. Separate values from independent digitizations were unavailable; therefore, retrospective inter-rater agreement could not be calculated.

### 2.4 Risk-of-bias assessment

Two reviewers independently assessed the risk of bias using the Systematic Review Centre for Laboratory Animal Experimentation (SYRCLE) tool (Hooijmans et al., 2014). The 10 domains were: sequence generation, baseline characteristics, allocation concealment, random housing, blinding of caregivers and investigators, random outcome assessment, blinding of outcome assessors, incomplete outcome data, selective outcome reporting, and other potential sources of bias. Each domain was rated as low, high, or unclear risk. A low-risk rating required sufficient information to support the relevant safeguard; insufficient reporting was rated as unclear, and an explicit method likely to introduce systematic bias was rated as high. A generic statement that animals were randomly allocated without specifying a sequence generation method was insufficient for a low-risk rating. Disagreements were resolved through discussion and, when necessary, through consultation with a third reviewer.

### 2.5 Effect-size calculation and meta-analysis

Continuous outcomes were summarized as Hedges’ g standardized mean differences (SMDs) between eligible *Epimedium*-derived interventions and untreated disease-model controls. Positive values indicate higher values after treatment; for Tb. Sp, DPD, PTH, TRACP-5b, and ALP, negative values may indicate improvement, depending on the model and assay. Inverse-variance random-effects models were used because heterogeneity in model, preparation, dose, treatment duration, site, and assay was expected *a priori*. Between-comparison variance was estimated by the DerSimonian–Laird method; heterogeneity was summarized using τ^2^, Cochran’s Q, and I^2^. When several eligible treatment arms shared one control group, the control group sample size was divided equally among the comparisons while the reported mean and SD were retained. This pragmatic correction reduces double counting, but does not remove within-study correlation. Outcomes were pooled only when at least two independent studies were available. Analyses were performed using a Python 3 workflow based on a standardized workbook; *p*-values were two-sided, and *p* < 0.05 was considered statistically significant. Prediction intervals were calculated for pooled outcomes. Because splitting a shared control adjusts weights but does not remove correlation, femoral BMD was additionally examined using one comparison per study (highest dose, with the latest time point used to break ties) and a three-level model with study-cluster robust inference.

Femoral BMD was selected as the principal outcome because it had the broadest evidence base and represented an anatomically defined skeletal site. Robustness was examined by sequentially removing all comparisons from one study ID. The funnel plot was treated as exploratory, and no formal asymmetry test was used because fewer than 10 independent studies contributed to the primary outcome. Additional exploratory analyses examined preparation type, OVX versus non-OVX models, treatment duration (≤8 versus >8 weeks), log10 dose, and the influence of the Qi 2019 (Qi et al., 2019) estimate. The risk of bias was assessed directly from each source report using the 10-domain SYRCLE tool; a statement of random allocation without an identifiable random component was rated as unclear. A *post hoc* lower-risk sensitivity analysis required low risk for both blinded outcome assessment and incomplete outcome data. Only Li et al. (2014) met this rule, so a pooled lower-risk sensitivity analysis was not estimable. Outcomes supported by three or fewer independent studies were explicitly treated as exploratory. Prediction intervals were reported where at least three independent studies permitted their estimation and meaningful interpretation; because such intervals are highly unstable at very small k, no prediction interval was interpreted for outcomes supported by only two studies. The certainty appraisal was conducted *post hoc* using an adaptation of GRADE for preclinical animal evidence. Each outcome started with a high level of certainty, with downgrading considered separately for risk of bias, inconsistency, indirectness, imprecision, and publication bias; no upgrading factors were applied (Hooijmans et al., 2018).

## 3 Results

### 3.1 Search results

The database search identified 1,654 records. After removal of 989 duplicates, 665 unique records underwent title and abstract screening, of which 533 were excluded. The full texts of 132 reports were assessed. After 103 full-text exclusions for the reasons shown in Figure 1, 29 independent rodent studies were retained. The study selection process is presented in the PRISMA flow diagram (Figure 1).

**FIGURE 1:**
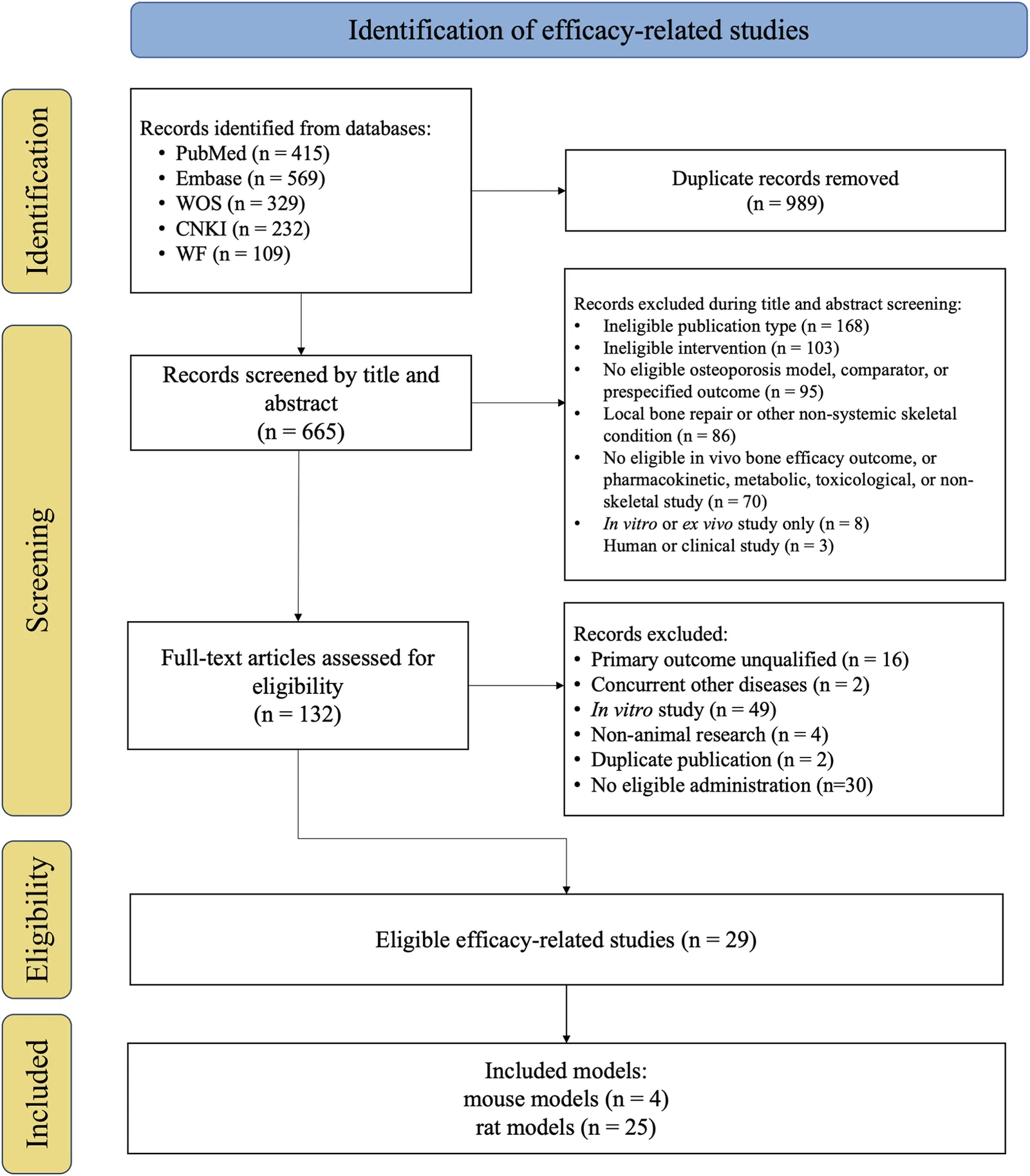
PRISMA 2020 flow diagram of study identification, screening, full-text eligibility assessment, and inclusion.

### 3.2 Study characteristics and risk of bias

The characteristics of the included studies are summarized in Table 1. The risk of bias was assessed for all 29 independent studies using the SYRCLE risk-of-bias tool, with study-level judgments and domain-level distributions presented in Figure 2. Only five reports explicitly identified the tested *Epimedium* species; accepted names, authorities, and family were checked against Plants of the World Online and are reported in Table 1 and Supplementary Table S3. The majority of preparations could not be authenticated retrospectively. Kang et al. (2013) included separable single-botanical and combination arms, but no outcome from this study appears in the eligible effect-size dataset; therefore, no combination data were pooled.

**TABLE 1:** Characteristics of the included animal studies.

Study ID	Animal	Intervention/preparation	Control n	Experimental n	Control	Experimental dose (mg/kg/d)	Duration	Bone-related outcomes
Ma et al. (1999)	Rats	Epimedium crude herb	10	6	Vehicle	2,500	8 w	BMD; TBV/TTV; Tb.Th; Tb.Sp; dynamic histomorphometry
Wang et al. (2000)	Rats	Epimedium	10	10	Vehicle	500	3 months	Lumbar BMD; IL-6 mRNA; E2
Ma et al. (2003)	Rats	Total flavonoids of Epimedium	6	6	Vehicle	50/100/200	28 days	BMD; Ca; P; ALP; BGP; PTH; DPD
Xu et al. (2004)	Rats	Epimedium pubescens flavonoids	10	10	Vehicle	300; DES 0.0225; combination	90 days	%Tb.Ar; Tb.Th; Tb.N; Ca; P; ALP; E2
Chen et al. (2004)	Rats	Total flavonoid extract of Epimedium sagittatum	8	8	Vehicle	50/100/200	3 months	BMD; biomechanics; ALP; OC; Ca/P; DPD
Bao et al. (2005)	Rats	Icariin	6	6	Vehicle	75/150/225	8 w	BMD; biomechanics; TRACP; ALP; BALP
Xie et al. (2005)	Rats	Herba Epimedii extract	8	9	Vehicle	110	3 months	Ca/P; ALP; DPD; Tb.Ar; Tb.Th; Tb.Sp; OPG/RANKL
Zhang et al. (2006)	Rats	Epimedium flavonoids	9	9	Vehicle	FE 10; Ca 56; FE + Ca	12 w	BMD; failure force; micro-CT; OC; DPD; PTH; Ca
Peng et al. (2008)	Rats	Epimedium-derived phytoestrogen	9	9	Vehicle	400	12 w	OC; DPD; BV/TV; Tb.N; Tb.Th; Tb.Sp; SMI; histomorphometry
Nian et al. (2009)	Rats	Icariin	10	10	Water	5/25/125	12 w	BMD; biomechanics; histomorphometry; Ca/P; ALP; E2; BGP
Chen et al. (2009)	Rats	Total flavone of Epimedium	20	20	Water	300	4 w	Lumbar BMD; OPG/OPGL mRNA
Peng et al. (2009)	Rats	Epimedium-derived flavonoids	10	10	Vehicle	10	4 months	BMD; micro-CT; static/dynamic histomorphometry; OC; TRACP5b
Wu et al. (2011)	Rats	Icariin	12	12	Vehicle	25/50/100	4 months	BMD; E2; ERβ mRNA in tibia/hypothalamic nuclei
Zheng et al. (2012)	Mice	Icariin	8	8	Vehicle	300	8 w	Micro-CT; bone formation/resorption histomorphometry; OPG dependence
Kang et al. (2013)	Rats	Epimedium/Ligustrum lucidum	10	10	Vehicle	Epimedium 9500; combination 4:3; raloxifene 6.25	3 w	Ca; P; AKP; StrACP; histopathology
Cao et al. (2014)	Rats	Total flavones of Epimedium leptorrhizum	18	17	Vehicle	700/1400/2,800	3 months	BMD; bone mineral; histomorphometry; PTH; PINP; ALP; Ca/P
Cheng et al. (2014)	Rats	Icariin	12	12	Water	ICA 25; genistein 10; ethinylestradiol 0.03	3 months	BMD; OC; TRACP5b; IL-6; VEGF; biomechanics; histology
Li et al. (2014)	Rats	Icariin	9	9	Vehicle	ICA 125; E2 0.025	12 w	BMD; marrow fat fraction; ALP/CTX; adipocyte parameters
Chen et al. (2016)	Rats	Icariin	14	14	Water	125/250/500; alendronate 5.04 mg/kg weekly	12 w	BMD; biomechanics; micro-CT; OPG/BGP; Wnt/β-catenin
Gao et al. (2017)	Rats	Epimedium brevicornum extract	10	10	Vehicle	60/120/240; E2 0.025	16 w	BMD; micro-CT; biomechanics; ALP; OC
Liu et al. (2018)	Rats	Water extract from Epimedium	12	12	Water	270/810/2,430	16 w	BMD; femoral strength; E2/BGP/OPG; neuropeptide signaling
He et al. (2018)	Rats	Icariin	6	6	Vehicle	25	4 w	BMD; micro-CT; biomechanics; TRACP5b; biological apatite
Luo et al. (2018)	Mice	Icariin	5	5	Saline	25	12 w	BV/TV; Tb.N; Tb.Sp; adipocytes; Wnt/β-catenin
Jing et al. (2019)	Mice	Icariin	10	10	PBS	100	2 months	Micro-CT; OC/ALP/TRACP5b/CTX; oxidative stress
Qi et al. (2019)	Rats	Icariin	10	10	Water	100	8 w	BMD; histomorphometry; ALP/CTX/TRACP5b/OC/PINP; OPG/RANKL/RUNX2
Zhou et al. (2021)	Rats	Icariin-containing diet	12	12	Phytoestrogen-free diet	50/500/3000 ppm; E2/raloxifene 1	12 w	BMD; cortical porosity; PINP/DPD; IGF-I/ERα
Mao et al. (2022)	Rats	Low-glycoside Epimedium flavonoids	6	6	Vehicle	35/70/140; XLGB 378	8 w	BMD; micro-CT; biomechanics; Ca/P/PINP/OC; BMP2/Runx2/Osterix
Cheng et al. (2022)	Rats	Icariin	8	8	Saline	600	6 w	ALP/NTX/Ca/P/Mg; TRAP; biomechanics; micro-CT; RANKL–p38/ERK–NFAT
Wang et al. (2025)	Mice	Dietary icariin	8	8	Control diet	2.5/10	14 months	Histology; PTGS2/HMOX1; ferroptosis-related transcriptomics/proteomics

Doses are expressed as mg/kg/day unless otherwise stated. The combination and positive control arms are listed for context, but were excluded from the quantitative synthesis when they did not meet the eligibility criteria. ICA, icariin; TFE, total flavonoids of *Epimedium*; EBME, *Epimedium* brevicornum extract; YYH-C, total flavonoids of *Epimedium* leptorrhizum; LG, low-glycoside *Epimedium* flavonoids; EPI, *Epimedium* water extract; FE/EF, *Epimedium* flavonoids; HEP, Herba Epimedii extract; BMD, bone mineral density; BV/TV, bone volume fraction; Tb.N, trabecular number; Tb.Th, trabecular thickness; Tb. Sp, trabecular separation. Full accepted botanical names are reported when the source study identifies them; otherwise, the species is recorded as not reported. *Epimedium brevicornu* Maxim., *Epimedium sagittatum* (Siebold & Zucc.) Maxim., *Epimedium pubescens* Maxim., and *Epimedium koreanum* Nakai (Berberidaceae) are national-pharmacopoeial origins; *Epimedium wushanense* T.S.Ying is designated for *Epimedii Folium Wushanensis*. *E. leptorrhizum* Stearn is a provincial standard but not a national pharmacopoeial origin. NR, not reported.

**FIGURE 2:**
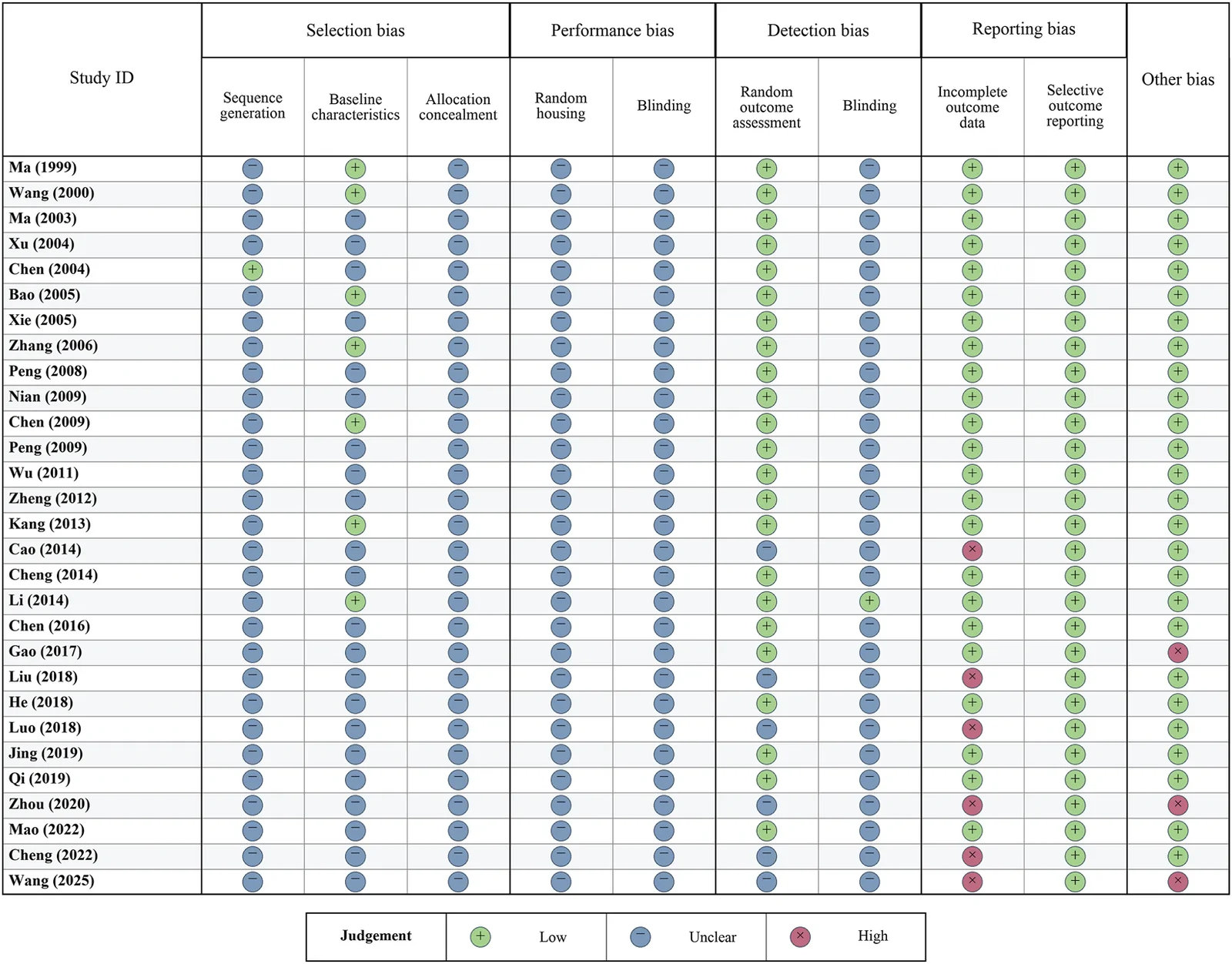
Study-level SYRCLE risk-of-bias assessment across 29 studies and 10 domains. Green (+), low risk; blue (−), unclear risk; red (×), high risk. Judgments were based on the information reported in each source article; absence of reporting was rated as unclear rather than low risk.

Overall, the risk-of-bias assessment yielded 110 low-risk judgments (37.9%), 171 unclear judgments (59.0%), and 9 high-risk judgments (3.1%) across 29 studies and 10 domains. The majority of uncertainty arose because methodological safeguards were not reported; the absence of reporting was rated as unclear rather than low risk.

Only Chen et al. (2004) described an adequate sequence-generation method (random-number method), and only Li et al. (2014) explicitly reported blinded outcome assessment. Baseline characteristics were rated as low risk in seven studies and as unclear in 22. Allocation concealment, random housing, and caregiver/investigator blinding were unclear in all 29 studies. Random outcome assessment was low risk in 23 studies and unclear in six.

Incomplete outcome data were considered low risk in 23 studies and high risk in six (Cao et al., 2014; Liu et al., 2018; Luo et al., 2018; Zhou et al., 2021; Cheng et al., 2022; and Wang et al., 2025) because the reported denominators decreased or conflicted with the allocated group sizes without an adequate explanation. Methods-to-results concordance supported a low risk of selective outcome reporting, although prospective protocols were unavailable. Other bias was low risk in 26 studies and high risk in Gao et al. (2017), Zhou et al. (2021), and Wang et al. (2025) because of irreconcilable dose or denominator/group-flow reporting. A *post hoc* lower-risk rule required low risk for both blinded outcome assessment and incomplete outcome data; only Li et al. (2014) met this rule, so a pooled lower-risk sensitivity analysis was not estimable.

### 3.3 Meta-analysis results

#### 3.3.1 Bone mineral density

The effects of Epimedium, icariin, and related preparations on BMD differed by anatomical site (Figure 3). For femoral BMD, 20 comparisons from 9 studies yielded SMD = 1.66 (95% CI: 1.07–2.25; 95% PI: −0.61 to 3.93; I^2^ = 73.77%). Qi et al. (2019) reported approximately 108 ± 6 versus 203 ± 9 mg/cm^3^ (model versus icariin; n = 10/group; mean ± SD), yielding g = 11.90. Inspection of the original figure and caption confirmed the values, error convention, and sample sizes; excluding this study reduced the estimate to 1.44 (95% CI: 0.96–1.92; 95% PI: −0.21 to 3.09; I^2^ = 60.35%). Lumbar BMD (5 comparisons, 3 studies; SMD = 2.07, 95% CI: 0.63–3.50; 95% PI: −1.12 to 5.25) and vertebral BMD (8 comparisons, 3 studies; SMD = 1.52, 95% CI: 0.70–2.35; 95% PI: −0.57 to 3.61) favored treatment, but these results were exploratory. Whole-body BMD was not significant (6 comparisons, 2 studies; SMD = 0.29, 95% CI: −0.21 to 0.80) and was also exploratory; a prediction interval was not interpreted at k = 2. These pooled values are average effects and should not be interpreted as the effect expected in a new study.

**FIGURE 3:**
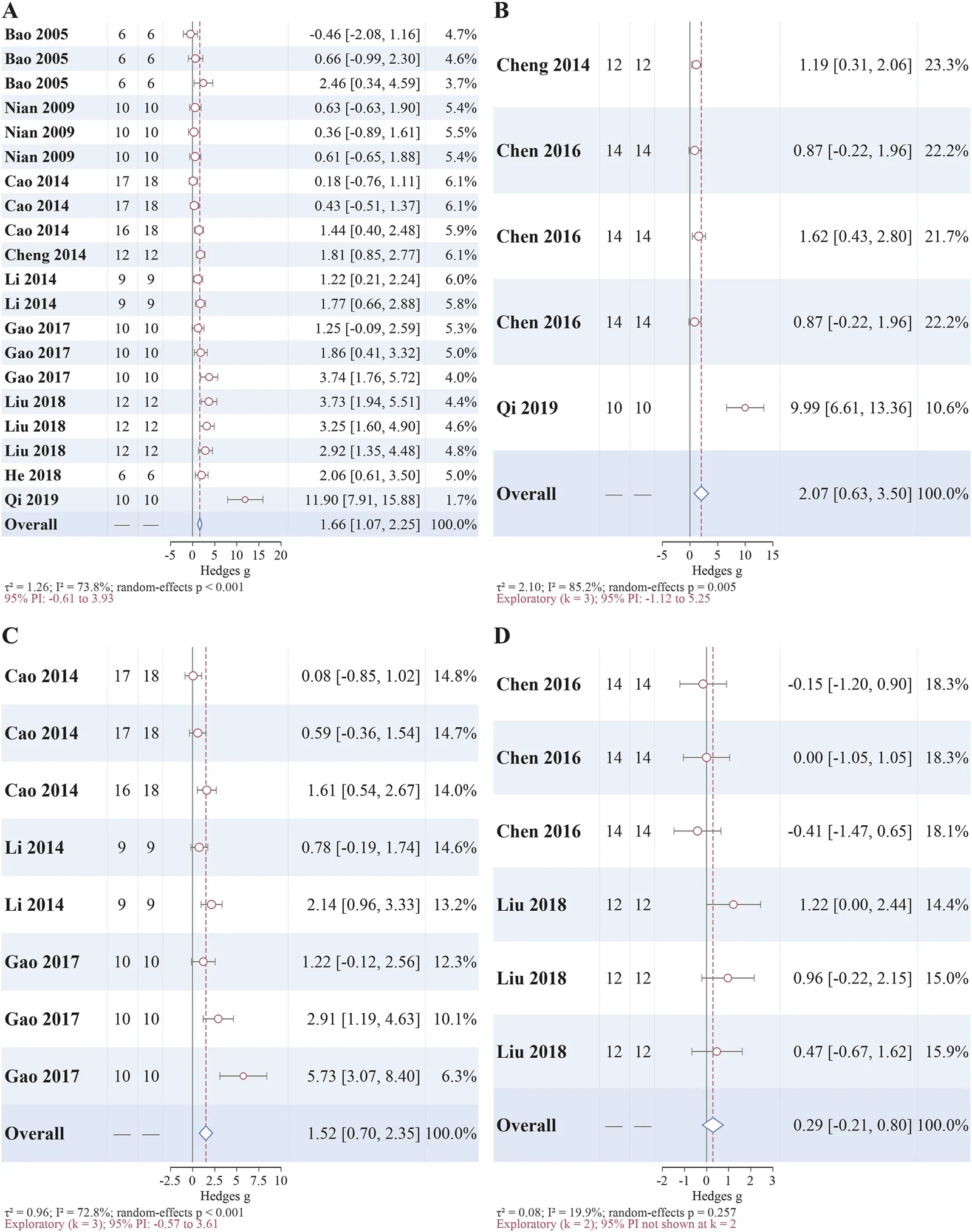
Effects of *Epimedium* and related preparations on bone mineral density by anatomical site. **(A)** Femoral BMD; **(B)** lumbar BMD; **(C)** vertebral BMD; and **(D)** whole-body BMD. Squares show Hedges’ g for eligible intervention arms with 95% CIs; diamonds show random-effects pooled estimates. Exp N, experimental-group sample size; Ctrl N, disease-model control sample size; CI, confidence interval; BMD, bone mineral density. ICA-L/M/H, low-/medium-/high-dose icariin; TFE-L/M/H, low-/medium-/high-dose total *Epimedium* flavonoids; EBME-L/M/H, low-/medium-/high-dose *Epimedium* brevicornum Maxim. extract; YYH-C, total flavonoids of *Epimedium* leptorrhizum; LG-L/M/H, low-/medium-/high-dose low-glycoside Epimedii Folium flavonoids; EPI, *Epimedium* water extract; FE, *Epimedium*-derived flavonoids; HEP, Herba Epimedii extract. Prediction intervals for the pooled estimates are reported in the manuscript and Supplementary Table S4.

#### 3.3.2 Trabecular microarchitecture

The pooled results for trabecular microarchitecture are summarized in Figure 4. The average effects favored treatment for BV/TV (9 comparisons, 7 studies; SMD = 3.27, 95% CI: 1.99–4.55; 95% PI: −0.18 to 6.72), Tb.N (12 comparisons, 8 studies; SMD = 1.70, 95% CI: 0.98–2.42; 95% PI: −0.37 to 3.77), Tb. Sp (16 comparisons, 10 studies; SMD = −3.44, 95% CI: −4.49 to −2.40; 95% PI: −7.10 to 0.21), and Tb.Th (19 comparisons, 11 studies; SMD = 1.99, 95% CI: 1.29–2.69; 95% PI: −0.71 to 4.69). Preparation-stratified Tb.Th estimates were 3.33 for purified icariin, 1.79 for flavonoid fractions, and 0.68 for crude/characterized extracts (interaction *p* = 0.035). These sparse, study-confounded subgroups are exploratory and do not establish comparative efficacy.

**FIGURE 4:**
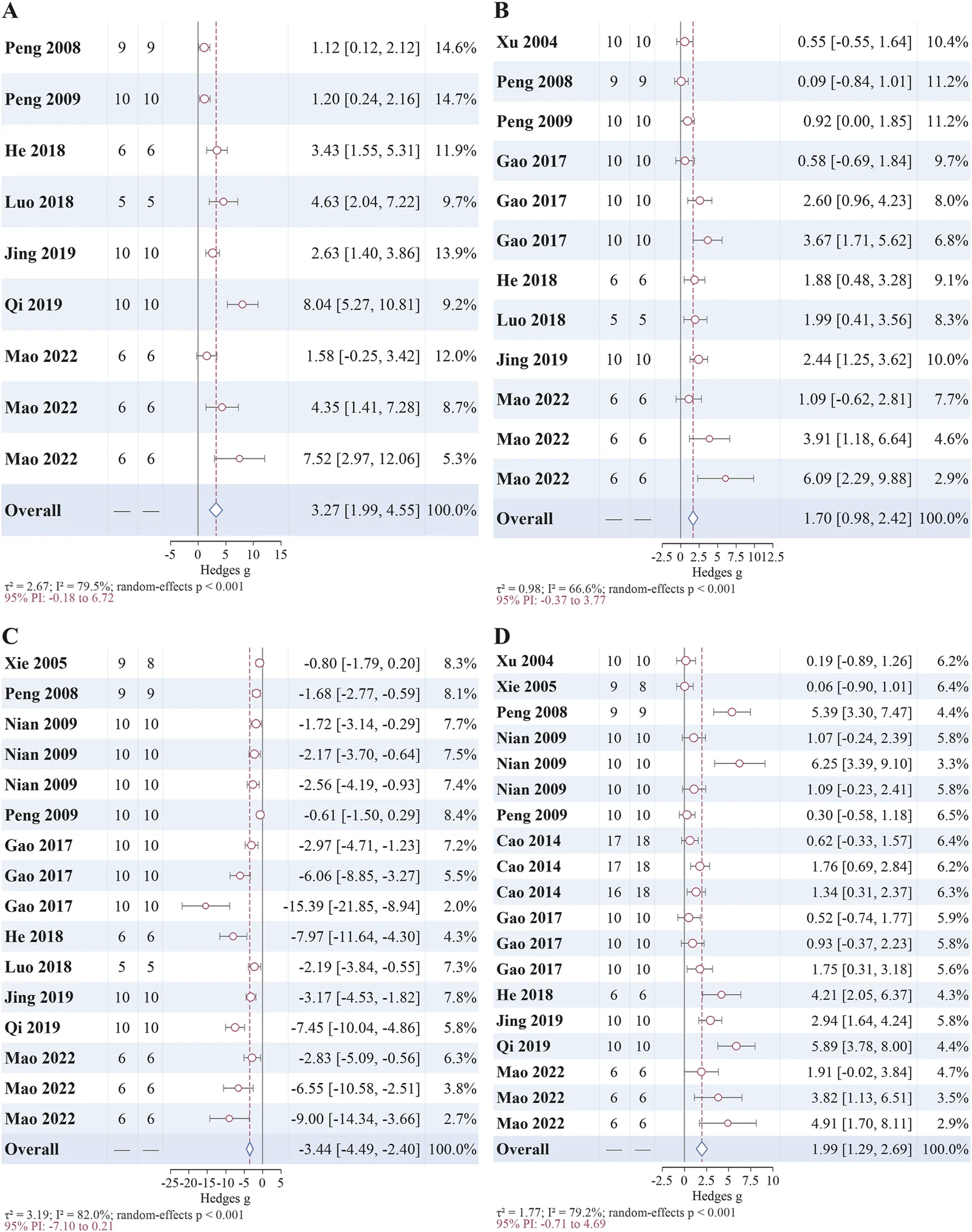
Effects of *Epimedium* and related preparations on trabecular microarchitecture. **(A)** bone volume fraction (BV/TV); **(B)** trabecular number (Tb.N); **(C)** trabecular separation (Tb.Sp); and **(D)** trabecular thickness (Tb.Th). Positive SMDs favor treatment for BV/TV, Tb.N, and Tb.Th; negative SMDs favor treatment for Tb. Sp. Exp N, experimental-group sample size; Ctrl N, disease-model control sample size; CI, confidence interval; SMD, standardized mean difference.

#### 3.3.3 Circulating mineral and bone-turnover markers

The pooled effects on circulating mineral and bone-turnover markers are summarized in Figure 5 and Table 2. Serum calcium (21 comparisons, 10 studies; SMD = 0.92, 95% CI: 0.40–1.45), phosphorus (20 comparisons, 9 studies; SMD = 1.37, 95% CI: 0.68–2.05), and estradiol (11 comparisons, 5 studies; SMD = 0.80, 95% CI: 0.42–1.18) were higher after treatment. ALP (22 comparisons, 11 studies; SMD = −1.19, 95% CI: −1.91 to −0.48), osteocalcin (9 comparisons, 5 studies; SMD = −2.59, 95% CI: −4.62 to −0.55), PTH (7 comparisons, 3 studies; SMD = −1.63, 95% CI: −2.16 to −1.10), and TRACP-5b (3 comparisons, 3 studies; SMD = −2.81, 95% CI: −3.52 to −2.10) were lower. PINP was not significant (7 comparisons, 3 studies; SMD = 0.65, 95% CI: −0.58 to 1.89; 95% PI: −2.51 to 3.81). Three-study outcomes are exploratory. Marker direction is phase- and assay-dependent; these data do not demonstrate increased intestinal calcium absorption or selective antiresorptive action without osteoblast suppression.

**FIGURE 5:**
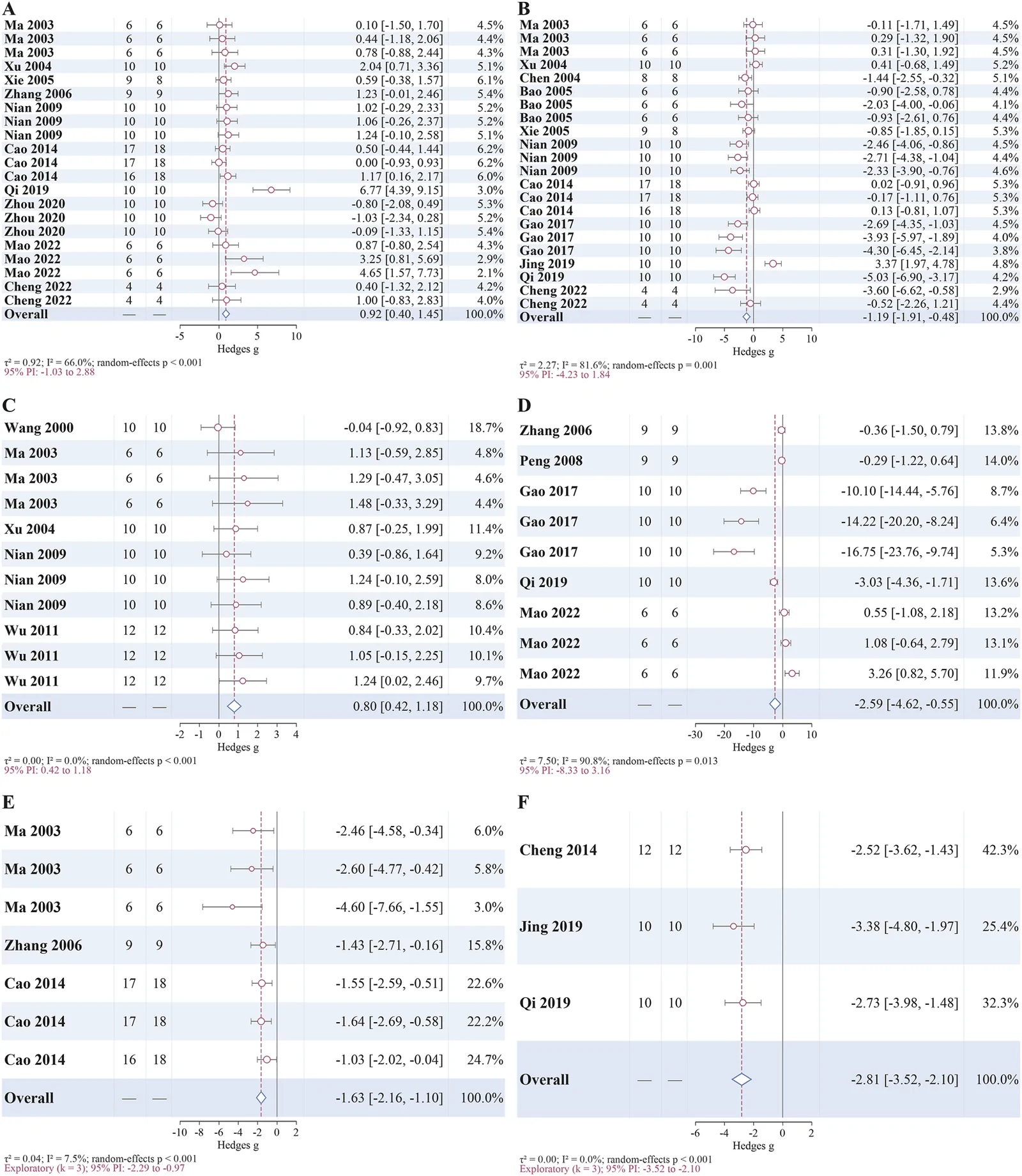
Effects of *Epimedium* and related preparations on circulating mineral and bone turnover markers. **(A)** Serum calcium; **(B)** alkaline phosphatase (ALP); **(C)** estradiol (E2); **(D)** osteocalcin (OC); **(E)** parathyroid hormone (PTH); and **(F)** tartrate-resistant acid phosphatase 5b (TRACP-5b). Lower ALP and OC relative to untreated osteoporotic controls may reflect attenuation of high-turnover remodeling and should not be interpreted as uniform suppression of osteoblast function. Exp N, experimental-group sample size; Ctrl N, disease-model control sample size; CI, confidence interval; SMD, standardized mean difference.

**TABLE 2:** Random-effects meta-analysis results for secondary biochemical, biomechanical, and histomorphometric outcomes.

Outcome	Comparisons	Studies	SMD	95% CI (95% PI)	I^2^ (%)
Serum calcium	21	10	0.92	0.40 to 1.45; PI −1.03 to 2.88	66.0
Phosphorus	20	9	1.37	0.68 to 2.05; PI −1.24 to 3.97	76.2
Estradiol	11	5	0.80	0.42 to 1.18; PI 0.42 to 1.18	0.0
ALP	22	11	−1.19	−1.91 to −0.48; PI −4.23 to 1.84	81.6
Osteocalcin	9	5	−2.59	−4.62 to −0.55; PI −8.33 to 3.16	90.8
PTH	7	3	−1.63	−2.16 to −1.10; PI −2.29 to −0.97	7.5
TRACP-5b	3	3	−2.81	−3.52 to −2.10; PI −3.52 to −2.10	0.0
Maximum load	9	4	2.34	1.29 to 3.39; PI −0.24 to 4.92	63.0
Elastic modulus	5	3	2.66	1.41 to 3.91; PI 0.32 to 5.01	53.7
SMI	3	3	−3.36	−6.31 to −0.41; PI −8.76 to 2.03	90.1
Trabecular area	5	3	1.59	0.49 to 2.70; PI −0.86 to 4.05	79.3
PINP	7	3	0.65	−0.58 to 1.89; PI −2.51 to 3.81	83.7

Effects are Hedges g standardized mean differences comparing the eligible *Epimedii Folium* treatment arm with the untreated disease-model control. Positive effects generally indicate higher values in the treatment group. For Tb. Sp, SMI, PTH, TRACP-5b, and other resorption or high-turnover indices, a negative effect may indicate a beneficial outcome. For estradiol and TRACP-5b, the DerSimonian–Laird variance estimate was τ^2^ = 0, so the normal-approximation PIs equal the corresponding CIs. This does not establish homogeneity, particularly with few studies. CI, confidence interval; I^2^, inconsistency statistic; SMD, standardized mean difference. Prediction intervals are shown in the same column as confidence intervals. Outcomes supported by three or fewer independent studies are exploratory. PI, prediction interval.

#### 3.3.4 Biomechanical and histomorphometric outcomes

The effects on biomechanical and histomorphometric outcomes are summarized in Figure 6 and Table 2. Maximum load was higher (9 comparisons, 4 studies; SMD = 2.34, 95% CI: 1.29–3.39; 95% PI: −0.24 to 4.92). Elastic modulus (5 comparisons, 3 studies; SMD = 2.66, 95% CI: 1.41–3.91; 95% PI: 0.32–5.01). Although the elastic modulus PI excluded zero, it should be interpreted with extreme caution because only three independent studies were available. SMI (3 comparisons, 3 studies; SMD = −3.36, 95% CI: −6.31 to −0.41; 95% PI: −8.76 to 2.03) and trabecular area (5 comparisons, 3 studies; SMD = 1.59, 95% CI: 0.49–2.70; 95% PI: −0.86 to 4.05) were exploratory. The average direction was compatible with improved *ex vivo* mechanical competence and trabecular structure; however, small evidence bases and wide PIs limit confidence.

**FIGURE 6:**
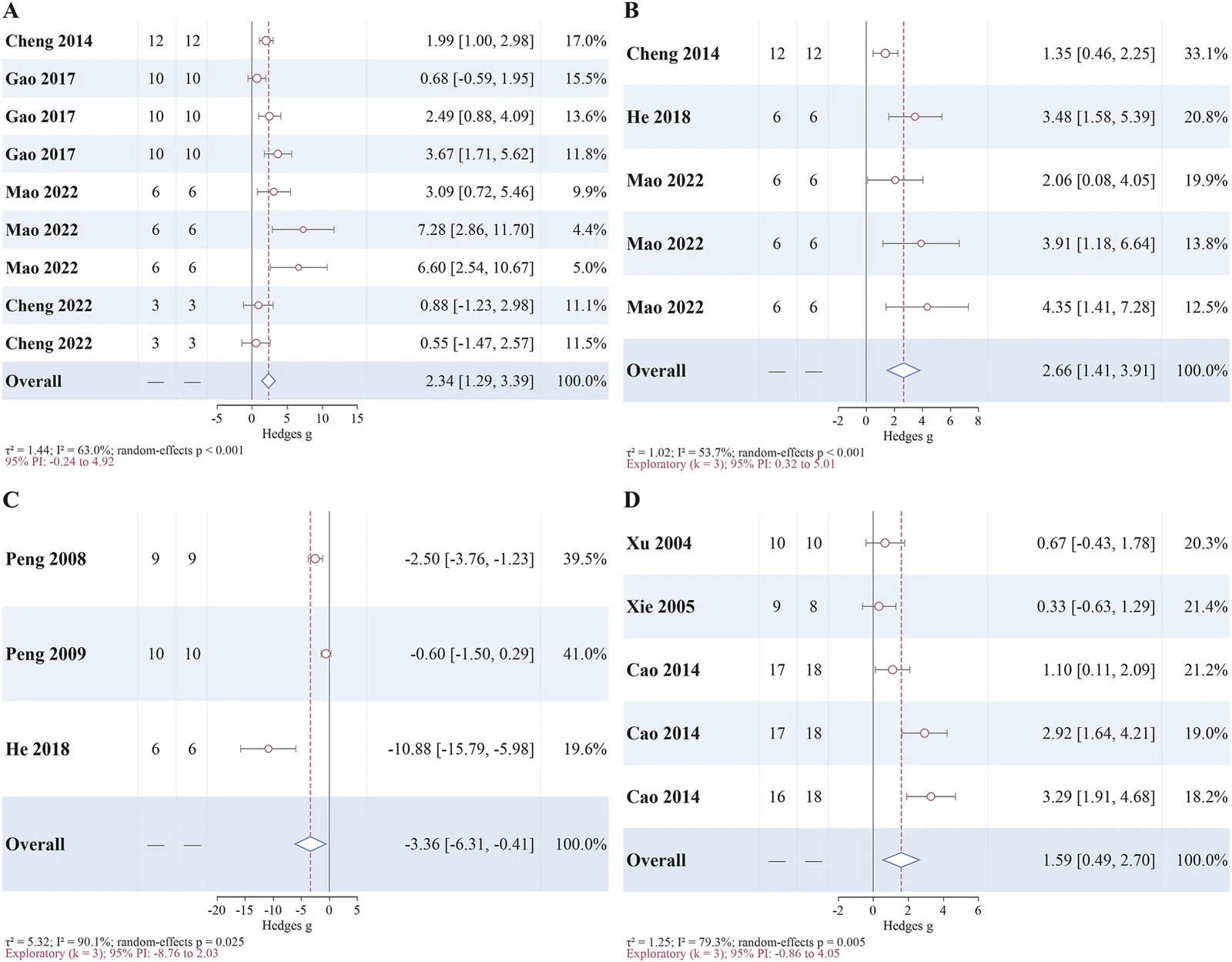
Effects of *Epimedium* and related preparations on biomechanical and histomorphometric outcomes. **(A)** Maximum load; **(B)** elastic modulus; **(C)** structure model index (SMI); and **(D)** trabecular area. Positive SMDs favor treatment for maximum load, elastic modulus, and trabecular area; negative SMDs favor treatment for SMI. Exp N, experimental-group sample size; Ctrl N, disease-model control sample size; CI, confidence interval; SMD, standardized mean difference.

### 3.4 Sensitivity analysis and exploratory small-study-effect assessment

Sensitivity and moderator analyses for femoral BMD are shown in Supplementary Table S4 and Supplementary Figure S2. Excluding Qi et al. (2019) reduced the SMD from 1.66 to 1.44. Selecting one comparison per study yielded an SMD of 2.40 (95% CI: 1.38–3.41; 95% PI: −0.36 to 5.15), and a three-level model with study-cluster robust inference yielded an SMD of 2.25 (95% CI: 0.40–4.10). Six of the nine femoral BMD studies contributed multiple effects. The preparation-stratified estimates were 1.47 (95% CI: 0.62–2.31; 11 comparisons, 6 studies) for purified icariin, 0.65 (95% CI: −0.08–1.39; 3 comparisons, 1 study) for flavonoid fractions, and 2.66 (95% CI: 1.81–3.51; 6 comparisons, 2 studies) for crude/characterized extracts (interaction *p* = 0.033). These strata were sparse and study-confounded. OVX versus non-OVX (*p* = 0.548) and ≤8 versus >8 weeks (*p* = 0.516) did not differ.

An exploratory study-cluster robust meta-regression analysis did not detect an association with dose per 10-fold increase (β = 0.64, 95% CI: −0.76 to 2.05; *p* = 0.304) or duration per 8 weeks (β = −1.48, 95% CI: −6.58 to 3.62; *p* = 0.505). These analyses were underpowered, and nominal mg/kg doses are not chemically comparable across purified icariin, fractions, and extracts. The nine-study funnel plot remains descriptive: asymmetry cannot distinguish reporting bias from the extreme Qi et al. (2019) estimate, heterogeneity, or chance. In Supplementary Figure S1A, the dashed line denotes the primary comparison-level DL estimate (SMD = 1.66); Supplementary Figure S1B displays the nine observations retained in the one-comparison-per-study analysis and is centered on its corresponding DL estimate (SMD = 2.40).

## 4 Discussion

### 4.1 Major findings and potential mechanisms

This systematic review and meta-analysis synthesized 29 independent rodent studies. Orally administered *Epimedium*, icariin, and related preparations generally improved regional BMD, trabecular microarchitecture, biomechanical competence, and selected bone-turnover indices. Compared with previous evidence syntheses, this review restricted eligibility to oral or intragastric administration and integrated site-specific BMD, microcomputed tomography, and biomechanical, histomorphometric, and biochemical outcomes (Ineichen et al., 2024; Shi et al., 2022). The principal prior quantitative benchmark was the 2016 seven-study icariin/OVX meta-analysis; the broader scope and expanded robustness analyses distinguish the present review (Xu et al., 2016). Nevertheless, the majority of prediction intervals crossed the null, so the pooled SMDs represent averages across heterogeneous experiments rather than the expected effects in new studies.

Femoral, lumbar, and vertebral BMD increased, whereas whole-body BMD did not. This site dependence is plausible because the femur and vertebral bodies contain metabolically responsive trabecular compartments, whereas whole-body densitometry averages regions with different cortical-to-trabecular compositions (Hu et al., 2024; Bittner-Frank et al., 2024; Wu et al., 2024).

The BMD findings were reinforced by higher bone volume fraction, trabecular number, and trabecular thickness; lower trabecular separation; and improvements in maximum load and elastic modulus. This agreement across imaging and mechanical outcomes is more persuasive than any single biomarker. Serum calcium and estradiol increased, whereas ALP, osteocalcin, PTH, and TRACP-5b decreased. Bone-turnover markers depend on growth stage, remodeling phase, sampling time, assay platform, feeding status, and time from model induction (Bhattoa et al., 2025; Filella and Guañabens, 2024; Martiniakova et al., 2024). In high-turnover ovariectomized controls, lower ALP and osteocalcin may indicate partial normalization of coupled remodeling, but assay heterogeneity precludes a universal interpretation. PINP was not significant, and lower ALP or osteocalcin could also indicate osteoblast suppression, assay variation, or phase-dependent sampling. Without consistent dynamic histomorphometry and paired formation/resorption markers, selective antiresorptive action cannot be inferred. The higher pooled serum calcium is a circulating concentration difference, not evidence of increased intestinal absorption; this is compatible with that of Zhang et al. (2006), who reported a skeletal effect independent of intestinal calcium absorption.

Mechanistic evidence is compatible with, but does not prove, the pooled *in vivo* effects. Icariin activated Wnt/β-catenin signaling and reduced sclerostin in bone marrow mesenchymal stem cells (Gao et al., 2021); in an ovariectomized model, SOST promoter methylation was associated with lower sclerostin and a shift from adipogenesis to osteogenesis (Chen et al., 2025). Other reports implicated SPI1/SMAD5 (Zhang et al., 2024a), Hippo-YAP/TAZ (Lin et al., 2025), and GLI1 (Xia et al., 2023). These pathways converge on lineage commitment and osteoblast function, but the majority were evaluated in isolated systems or single models rather than as mediators of the pooled outcomes. These pathways were not consistently measured across the included studies, and the data were too sparse for meaningful vote counting; they are presented as supporting biological context rather than as mechanisms established by this meta-analysis.

The lower TRACP-5b concentration and improved trabecular architecture are also consistent with reduced osteoclastogenesis. RANKL–RANK signaling activates TRAF6, NF-κB, MAPKs, c-Fos, and NFATc1 (Boyce et al., 2023). Experimental reports linked icariin to Cullin 3/Nrf2/OH signaling (Si et al., 2024), the suppression of the RANKL–p38/ERK–NFAT pathway (Cheng et al., 2022), and ERα/c-Src/RANK signaling (Yang et al., 2024). Baohuoside I, an intestinally generated metabolite of icariin, has similarly inhibited MAPK- and NF-κB-related osteoclastogenic signaling in ovariectomized mice (Ma et al., 2022). Parent glycosides and active metabolites may therefore both contribute after oral administration.

Autophagy is another potential modifier of osteoblast and mesenchymal stem cell homeostasis. Icariin activated autophagy-associated signaling in ovariectomized rats (Zou et al., 2024) and restored osteogenic differentiation in a ketogenic diet model through mTOR/autophagy regulation (Liu et al., 2024). Because autophagy depends on cell type, dose, and disease stage, these observations are context-specific rather than evidence of a universal mechanism (Chen et al., 2024a).

Oxidative injury offers a link between impaired osteogenesis and increased resorption. Iron overload-induced lipid peroxidation can trigger osteoblast ferroptosis (Jiang et al., 2022), and dietary icariin modified iron handling and ferroptosis-related proteins in an age-related model (Wang et al., 2025). Oxidative and inflammatory signals also reinforce one another across osteoporosis models (Luo et al., 2025). These associations do not establish that ferroptosis mediates the pooled structural outcomes.

The focus on oral administration also makes intestinal metabolism relevant. In ovariectomized rats, icariin altered gut microbial and metabolite profiles alongside changes in bone loss (Wang et al., 2022). Short-chain fatty acids, bile acids, and tryptophan metabolites can affect calcium handling, barrier function, inflammation, and osteoclast differentiation (Lyu et al., 2023). The activity of orally administered *Epimedium* glycosides may, therefore, depend partly on microbial conversion and host metabolism (Zhang et al., 2024b), although this was not tested as a mediator in the included studies.

Model-specific mechanisms may contribute to heterogeneity. *Epimedium* brevicornum extract modified AGE–RAGE-related metabolic and inflammatory signaling in diabetic osteoporosis (Lei et al., 2025), whereas epimedin B was associated with the OPG/RANKL balance in another model of diabetes (Zhang et al., 2025). Oxidative stress is shared across several high-risk states (Zhang et al., 2023b), but estrogen deficiency, diabetes, disuse, toxicant exposure, and genetic OPG deficiency have distinct pathophysiology and should not be assumed to share a single mechanism.

The leave-one-study-out analysis showed that no single study reversed the femoral BMD finding. However, visual funnel-plot asymmetry and one unusually large study-level effect could reflect small-study effects, selective reporting, underestimated variance, or true pharmacological heterogeneity. The leave-one-study-out direction was unchanged, but this analysis does not address residual dependence, selective reporting, or other risks of bias; the magnitude therefore remains uncertain. The Qi et al. (2019) value arose from a large mean difference relative to small reported SDs rather than a transcription or SEM/SD error. Excluding Qi et al. (2019) and using dependence-aware models retained a positive average estimate but widened uncertainty.

### 4.2 Future directions and clinical relevance

Human evidence is encouraging but insufficient to define an osteoporosis regimen. A 6-week, placebo-controlled trial of a standardized *Epimedium* prenyl flavonoid extract primarily assessed the safety, pharmacokinetics, and surrogate bone markers rather than BMD or fractures (Yong et al., 2021). A clinical meta-analysis reported a possible benefit, but it included heterogeneous formulations, concomitant therapies, outcomes, and study quality (Li et al., 2024). These data support the need for further evaluation rather than declaring therapeutic equivalence to approved anti-fracture drugs. Preclinical studies should report the botanical identity, the part of the plant used, whether a voucher or commercial batch was utilized, the origin of the plant, how it was processed, the solvent used for extraction, the drug-to-extract ratio, and the results of quantitative chromatography (Tan et al., 2024); nominally similar extracts may be pharmacologically different (Chen et al., 2025). Exposure-response studies should measure parent glycosides and active metabolites alongside skeletal and safety outcomes (Fu et al., 2025). Study quality should be improved through prespecified sample sizes, explicit randomization and allocation concealment, blinded assessments, and complete accounting of animals (Percie du Sert et al., 2020). Established osteoporosis drugs have demonstrated fracture-risk reduction, whereas *Epimedium*-derived products have not (Chen et al., 2024b). Future reports should also state the full accepted species name with authority and family, pharmacopoeial status, raw or processed state, processing method, and quantitative marker profile in accordance with ConPhyMP principles (Heinrich et al., 2022; Zhang et al., 2022; He et al., 2020). Species-level chemical differences may contribute to heterogeneity, but incomplete source reporting prevented formal analysis here.

A 24-month randomized trial in 100 late postmenopausal women provides useful context. The tested preparation contained icariin, daidzein, and genistein. A total of 85 participants completed follow-up, and BMD was maintained relative to a calcium-treated placebo group, but fracture outcomes were not assessed, and the design did not isolate icariin (Zhang et al., 2007). These limitations place the clinical signal at the proof-of-concept stage. Only Kang et al. (2013) described processed *Epimedii Folium*, without reporting the source species or processing method; no processed-versus-raw comparison was possible, and no outcome from that study was pooled. Processing should be treated as defining a distinct preparation in future studies (He et al., 2020).

For translation, the same chemically defined product should first be reproduced across laboratories and evaluated with exposure-based dose selection and parent–metabolite pharmacokinetics. The present mg/kg data should not be converted into a proposed human dose by body-surface-area scaling alone. Botanical extracts should be characterized in accordance with ConPhyMP principles (Heinrich et al., 2022). Short-term tolerability does not establish long-term safety; repeat-dose hepatic, renal, hematologic, cardiovascular, endocrine, and reproductive effects, along with clinically relevant herb-drug interactions, require specific evaluation. Short-term changes in BMD or turnover markers should not be presented as evidence of fracture prevention.

## 5 Limitations

Several limitations should be considered. Substantial heterogeneity across species, models, preparations, doses, treatment durations, skeletal sites, and assay methods reduced the precision of the pooled estimates. Purified icariin, flavonoid fractions, and botanical extracts are not chemically interchangeable, and random-effects models cannot fully account for this pharmacological variability. Botanical authentication, extraction yield, and marker-compound content were inconsistently reported, preventing stratification by preparation quality. Some studies contributed multiple intervention arms sharing a common control, leaving residual within-study correlation despite splitting the control sample size. Several outcomes, including whole-body BMD, structure model index, PTH, and TRACP-5b, were supported by few independent studies. The majority of prediction intervals crossed the null. Preparation subgroups were sparse and study-confounded, and the three-level robust analysis showed wider uncertainty than the primary model; these limitations restrict the interpretation of the pooled averages.

The majority of SYRCLE domains remained unclear because key methodological safeguards were insufficiently reported, and nine domain-level judgments were at high risk. Prospective protocols were unavailable; however, comparison of the published methods with the results did not identify any omitted prespecified outcomes, so selective outcome reporting was rated as low risk, while residual reporting bias remains possible. Graph-derived values accounted for 31.0% of comparisons; although every pooled graph-derived |g| > 5 was source-checked, separate digitizer-level records were unavailable for retrospective agreement analysis. The femoral BMD funnel plot was visually asymmetric, but only nine independent studies contributed, and one extreme estimate strongly influenced the distribution; therefore, it was considered exploratory and not interpreted as evidence of publication bias. Mechanistic pathways were not consistently measured across the pooled studies, precluding conclusions about pathway mediation.

Finally, regional BMD, microarchitectural, and *ex vivo* mechanical outcomes in rodents are surrogate endpoints. The included models do not reproduce the combined effects of aging, multimorbidity, polypharmacy, falls, sex, cortical porosity, and secondary osteoporosis etiologies on human fracture risk. Safety outcomes were not systematically synthesized.

Despite these limitations, concordant improvements in regional BMD, trabecular microarchitecture, biomechanical strength, and bone-remodeling markers support the osteoprotective potential of orally administered Epimedium-derived interventions. Further studies should use chemically standardized preparations, exposure-informed dosing, and rigorous experimental designs.

## 6 Conclusion

In rodent models of osteoporosis or pathological bone loss, random-effects pooled estimates generally favored the examined orally administered icariin and *Epimedium*-derived preparations for regional BMD, trabecular microarchitecture, and selected *ex vivo* biomechanical outcomes. The structural findings were directionally compatible with bone turnover and mechanistic evidence, but high heterogeneity, small sample sizes, chemical variability, incomplete blinding and allocation reporting, and limited exposure data reduce certainty in effect magnitude. These findings do not establish fracture prevention, an effective human dose, comparative efficacy, or clinical safety. Future work should use authenticated and quantitatively standardized preparations, exposure-linked dosing, preregistered and blinded designs, harmonized outcomes, and concurrent safety evaluations before clinical translation. The majority of prediction intervals crossed the null, and certainty for the principal outcomes was very low; accordingly, the pooled estimates should not be interpreted as the expected effects in a new experiment or as evidence of a common mechanism.

## Data Availability

The original contributions presented in the study are included in the article/Supplementary Material; further inquiries can be directed to the corresponding author.

## Glossary

AGE: Advanced glycation end product
ALP: Alkaline phosphatase
BMD: Bone mineral density
BMP: Bone morphogenetic protein
BMSC: Bone marrow mesenchymal stem cell
BV/TV: Bone volume fraction (bone volume/total volume)
CI: Confidence interval
CTX: C-terminal telopeptide of type I collagen
DPD: Deoxypyridinoline
E2: Estradiol
ER: Estrogen receptor
IGF-I: Insulin-like growth factor I
IL-6: Interleukin-6
MAPK: Mitogen-activated protein kinase
micro-CT: Micro-computed tomography
mRNA: Messenger RNA
mTOR: Mechanistic target of rapamycin
NF-κB: Nuclear factor kappa B
NFATc1: Nuclear factor of activated T cells 1
NOX: NADPH oxidase
Nrf2: Nuclear factor erythroid 2-related factor 2
NTX: N-terminal telopeptide of type I collagen
OC: Osteocalcin
OPG: Osteoprotegerin
OVX: Ovariectomy or ovariectomized
PI3K: Phosphoinositide 3-kinase
PINP: Procollagen type I N-terminal propeptide
PRISMA: Preferred Reporting Items for Systematic Reviews and Meta-Analyses
PROSPERO: International Prospective Register of Systematic Reviews
PTH: Parathyroid hormone
RAGE: Receptor for advanced glycation end products
RANK: Receptor activator of nuclear factor κB
RANKL: Receptor activator of nuclear factor κB ligand
ROS: Reactive oxygen species
RUNX2: Runt-related transcription factor 2
SD: Standard deviation
SEM: Standard error of the mean
SMI: Structure model index
SMD: Standardized mean difference
SOST: Sclerostin gene
SYRCLE: Systematic Review Centre for Laboratory Animal Experimentation
Tb.Ar: Trabecular area
Tb.N: Trabecular number
Tb.Sp: Trabecular separation
Tb.Th: Trabecular thickness
TRAP: Tartrate-resistant acid phosphatase
TRACP-5b: Tartrate-resistant acid phosphatase isoform 5b
VEGF: Vascular endothelial growth factor
