# Supplementary material for: Efficacy of Epimedii Folium and related preparations in experimental osteoporosis: a systematic review and meta-analysis
Source: Front Pharmacol. 2026 Oct 14;17:1951582. doi: 10.3389/fphar.2026.1951582 (PMC13713888; doi:10.3389/fphar.2026.1951582)
Supplement: Supplementary file 1 [file Supplementaryfile1.docx]

**Supplementary Material 1**

**Supplementary Table S1. Search strategy and working queries**

| **Search ID** | **Content** | **Query** |
| --- | --- | --- |
| Databases and date | Sources and limits | PubMed/MEDLINE, Embase, Web of Science Core Collection, China National Knowledge Infrastructure (CNKI), and Wanfang Data were searched from database inception to June 13, 2026. No language restriction was applied. |
| #1 | Epimedii Folium and preparations | ("Epimedium" OR "Epimedii Folium" OR "Herba Epimedii" OR "Yin Yang Huo" OR "Yinyanghuo" OR "icariin" OR "icaritin" OR "icariside II" OR "baohuoside I" OR "epimedin A" OR "epimedin B" OR "epimedin C" OR "Epimedium extract" OR "Epimedium flavonoids" OR "total flavonoids of Epimedium" OR "low-glycoside Epimedium flavonoids") |
| #2 | Target animals | ("rat" OR "rats" OR "mouse" OR "mice" OR "murine" OR "rodent" OR "rodents") |
| #3 | Osteoporosis / pathological bone loss | ("osteoporosis" OR "osteoporotic" OR "osteopenia" OR "bone loss" OR "pathological bone loss" OR "bone mineral density" OR "BMD" OR "bone microarchitecture" OR "bone metabolism" OR "ovariectomy" OR "ovariectomized" OR "OVX" OR "postmenopausal osteoporosis") |
| Search query: #1 AND #2 AND #3 | English databases | ("Epimedium" OR "Epimedii Folium" OR "Herba Epimedii" OR "Yin Yang Huo" OR "Yinyanghuo" OR "icariin" OR "icaritin" OR "icariside II" OR "baohuoside I" OR "epimedin A" OR "epimedin B" OR "epimedin C" OR "Epimedium extract" OR "Epimedium flavonoids" OR "total flavonoids of Epimedium" OR "low-glycoside Epimedium flavonoids") AND ("rat" OR "rats" OR "mouse" OR "mice" OR "murine" OR "rodent" OR "rodents") AND ("osteoporosis" OR "osteoporotic" OR "osteopenia" OR "bone loss" OR "pathological bone loss" OR "bone mineral density" OR "BMD" OR "bone microarchitecture" OR "bone metabolism" OR "ovariectomy" OR "ovariectomized" OR "OVX" OR "postmenopausal osteoporosis") |
| #4 | 淫羊藿及其制剂 | ("淫羊藿" OR "淫羊藿苷" OR "淫羊藿总黄酮" OR "淫羊藿黄酮" OR "淫羊藿提取物" OR "淫羊藿水提物" OR "淫羊藿低糖苷黄酮" OR "朝藿定A" OR "朝藿定B" OR "朝藿定C" OR "宝藿苷" OR "依卡瑞因" OR "异补骨脂苷") |
| #5 | 实验动物 | ("大鼠" OR "小鼠" OR "鼠" OR "啮齿动物" OR "动物" OR "动物模型") |
| #6 | 骨质疏松 / 病理性骨丢失 | ("骨质疏松" OR "骨量减少" OR "骨丢失" OR "骨密度" OR "骨小梁" OR "骨代谢" OR "骨质疏松模型" OR "去卵巢" OR "卵巢切除" OR "绝经后骨质疏松") |
| Search query: #4 AND #5 AND #6 | Chinese databases | ("淫羊藿" OR "淫羊藿苷" OR "淫羊藿总黄酮" OR "淫羊藿黄酮" OR "淫羊藿提取物" OR "淫羊藿水提物" OR "淫羊藿低糖苷黄酮" OR "朝藿定A" OR "朝藿定B" OR "朝藿定C" OR "宝藿苷" OR "依卡瑞因" OR "异补骨脂苷") AND ("大鼠" OR "小鼠" OR "鼠" OR "啮齿动物" OR "动物" OR "动物模型") AND ("骨质疏松" OR "骨量减少" OR "骨丢失" OR "骨密度" OR "骨小梁" OR "骨代谢" OR "骨质疏松模型" OR "去卵巢" OR "卵巢切除" OR "绝经后骨质疏松") |

**Supplementary Table S2. Inclusion and exclusion criteria**

| **Items** | **Criteria** |
| --- | --- |
| Inclusion criteria | 1) The experiment used an in vivo rat or mouse model of osteoporosis, osteopenia, or experimentally induced pathological bone loss. 2) The intervention was icariin, a single-herb Epimedii Folium preparation, or a separable Epimedium extract/flavonoid fraction. 3) The intervention was administered orally, intragastrically, by gavage, through diet, or by another enteral route. 4) A concurrent untreated or vehicle-treated disease-model control group was available. 5) At least one quantitative outcome related to BMD, trabecular microarchitecture, histomorphometry, bone biomechanics, or bone metabolism was reported. 6) Group sample size, mean, and dispersion were reported or could be extracted from tables, text, or figures. |
| Exclusion criteria | 1) In vitro-only, ex vivo-only, clinical, review, commentary, protocol, or meta-analysis publications were excluded. 2) Studies using species other than rats or mice, or healthy animals without an osteoporosis or pathological bone-loss model, were excluded. 3) Fracture repair, local bone defect, implant, or other non-systemic skeletal condition studies were excluded unless an eligible osteoporosis model was present. 4) Positive-control-only studies, non-enteral administration studies, and inseparable multi-herb combinations were excluded from quantitative synthesis. 5) Studies without a concurrent disease-model control group were excluded. 6) Duplicate or overlapping datasets were excluded; the most complete report was retained. 7) Studies without extractable quantitative data were excluded from the meta-analysis. |

Note: The search syntax was adapted to database-specific fields and indexing conventions. English databases used free-text terms and subject headings where available. Chinese databases used corresponding Chinese terms and free-text searches in title, abstract, and keyword fields where supported.

# **Supplementary Figure**

Supplementary Figure S1. Robustness and exploratory small-study-effect assessment for femoral BMD. (A) Leave-one-study-out random-effects estimates; the dashed line denotes the primary comparison-level DL estimate (SMD = 1.66). (B) Exploratory funnel plot of the nine observations retained in the one-comparison-per-study analysis (highest dose, with the latest time point used to break ties); the dashed line and funnel contours are centered on the corresponding study-level DL estimate (SMD = 2.40).

**Supplementary Table S3. Preparation, model, botanical identity, processing, and quantitative-use audit**

| Study | Preparation / model | Botanical identity / processing | Quantitative use |
| --- | --- | --- | --- |
| Ma 1999 (76) | Crude botanical drug / OVX | Species NR; processing NR | Included |
| Wang 2000 (77) | Crude/unspecified / OVX | Species NR; processing NR | Included |
| Ma 2003 (78) | Flavonoid fraction / OVX | Species NR; processing NR | Included |
| Xu 2004 (79) | Flavonoid fraction / OVX | E. pubescens Maxim.; raw/processed NR | Included |
| Chen 2004 (80) | Flavonoid fraction / OVX | E. sagittatum (Siebold & Zucc.) Maxim.; processing NR | Included |
| Bao 2005 (81) | Purified icariin / OVX | Botanical source NR | Included |
| Xie 2005 (82) | Crude/unspecified / OVX | Species NR; processing NR | Included |
| Zhang 2006 (83) | Flavonoid fraction / OVX | E. brevicornu Maxim.; processing NR | Included |
| Peng 2008 (84) | Flavonoid fraction / OVX | Species NR; processing NR | Included |
| Nian 2009 (85) | Purified icariin / OVX | Botanical source NR | Included |
| Chen 2009 (86) | Flavonoid fraction / iron overload | Species NR; processing NR | Included |
| Peng 2009 (87) | Flavonoid fraction / OVX | Species NR; processing NR | Included |
| Wu 2011 (88) | Purified icariin / OVX | Botanical source NR | Included |
| Zheng 2012 (89) | Purified icariin / OPG deficiency | Botanical source NR | Included |
| Kang 2013 (90) | Processed botanical drug / retinoic acid | Species/process NR; combination arm present | No outcome pooled |
| Cao 2014 (91) | Flavonoid fraction / retinoic acid | E. leptorrhizum Stearn; processing NR | Included |
| Cheng 2014 (92) | Purified icariin / OVX | Botanical source NR | Included |
| Li 2014 (93) | Purified icariin / OVX | Botanical source NR | Included |
| Chen 2016 (94) | Purified icariin / OVX | Botanical source NR | Included |
| Gao 2017 (95) | Crude/characterized extract / glucocorticoid | E. brevicornu Maxim.; processing NR | Included |
| Liu 2018 (96) | Crude/unspecified / OVX | Species NR; processing NR | Included |
| He 2018 (97) | Purified icariin / hindlimb suspension | Botanical source NR | Included |
| Luo 2018 (98) | Purified icariin / thioacetamide | Botanical source NR | Included |
| Jing 2019 (99) | Purified icariin / OVX | Botanical source NR | Included |
| Qi 2019 (100) | Purified icariin / diabetes | Botanical source NR | Included; source verified |
| Zhou 2020 (101) | Purified icariin / OVX | Botanical source NR | Included |
| Mao 2022 (102) | Flavonoid fraction / retinoic acid | Species NR; processing NR | Included |
| Cheng 2022 (49) | Purified icariin / thioacetamide | Botanical source NR | Included |
| Wang 2025 (56) | Purified icariin / ageing | Botanical source NR | Included |

NR, not reported. Accepted names and authorities were checked against Plants of the World Online; all named Epimedium taxa belong to Berberidaceae.

**Supplementary Table S4. Femoral BMD sensitivity and moderator analyses**

| Analysis | Evidence | Estimate (95% CI) | Comment |
| --- | --- | --- | --- |
| Primary DL model | 20 comparisons; 9 studies | 1.66 (1.07 to 2.25) | PI -0.61 to 3.93 |
| Excluding Qi 2019 (100) | 19 comparisons; 8 studies | 1.44 (0.96 to 1.92) | PI -0.21 to 3.09 |
| One comparison/study | 9 comparisons; 9 studies | 2.40 (1.38 to 3.41) | Highest dose; latest time used to break ties; PI -0.36 to 5.15 |
| Three-level cluster-robust | 20 comparisons; 9 studies | 2.25 (0.40 to 4.10) | REML; robust CI |
| Post hoc lower-risk rule | 2 comparisons; 1 study | Not pooled | Only Li 2014 (93) met the rule of low risk for both blinded outcome assessment and incomplete outcome data |
| Preparation: purified icariin | 11 comparisons; 6 studies | 1.47 (0.62 to 2.31) | Exploratory; interaction p = 0.033 |
| Preparation: flavonoid fraction | 3 comparisons; 1 study | 0.65 (-0.08 to 1.39) | Exploratory; interaction p = 0.033 |
| Preparation: crude/characterized extract | 6 comparisons; 2 studies | 2.66 (1.81 to 3.51) | Exploratory; interaction p = 0.033 |
| Preparation interaction | 3 classes | p = 0.033 | Sparse and study-confounded; no comparative efficacy inference |
| OVX vs non-OVX | 2 groups | p = 0.548 | Exploratory |
| ≤8 vs >8 weeks | 2 groups | p = 0.516 | Exploratory |
| Dose meta-regression | Per 10-fold dose | beta 0.64 (-0.76 to 2.05) | p = 0.304 |
| Duration meta-regression | Per 8 weeks | beta -1.48 (-6.58 to 3.62) | p = 0.505 |

**Supplementary Table S5. Multi-arm structure by outcome**

| Outcome | Comparisons | Studies | Studies with multiple effects | Maximum effects/study |
| --- | --- | --- | --- | --- |
| ALP | 22 | 11 | 6 | 3 |
| BGP/OC | 6 | 2 | 2 | 3 |
| BMD | 12 | 6 | 3 | 3 |
| BMD-femur | 20 | 9 | 6 | 3 |
| BMD-lumbar | 5 | 3 | 1 | 3 |
| BMD-vertebra | 8 | 3 | 3 | 3 |
| BMD-whole body | 6 | 2 | 2 | 3 |
| BV/TV | 9 | 7 | 1 | 3 |
| Ca | 21 | 10 | 6 | 3 |
| DPD | 4 | 2 | 1 | 3 |
| DPD/Cr | 2 | 2 | 0 | 1 |
| E2 | 11 | 5 | 3 | 3 |
| ES/BS | 2 | 2 | 0 | 1 |
| Elastic modulus | 5 | 3 | 1 | 3 |
| MAR | 2 | 2 | 0 | 1 |
| Maximum load | 9 | 4 | 3 | 3 |
| N.Oc/B.Pm | 2 | 2 | 0 | 1 |
| OC | 9 | 5 | 2 | 3 |
| OS/BS | 2 | 2 | 0 | 1 |
| P | 20 | 9 | 6 | 3 |
| PINP | 7 | 3 | 2 | 3 |
| PTH | 7 | 3 | 2 | 3 |
| SMI | 3 | 3 | 0 | 1 |
| TRACP5b | 3 | 3 | 0 | 1 |
| Tb.Ar | 5 | 3 | 1 | 3 |
| Tb.N | 12 | 8 | 2 | 3 |
| Tb.Sp | 16 | 10 | 3 | 3 |
| Tb.Th | 19 | 11 | 4 | 3 |

**Supplementary Table S6. Source-data verification**

| Item | Check | Conclusion |
| --- | --- | --- |
| Qi 2019 (100) femoral BMD | Figure 2 and caption: model approx. 108 ± 6; icariin approx. 203 ± 9 mg/cm³; n = 10/group; mean ± SD | g = 11.90 retained; no transcription or SEM/SD error |
| Graph extraction | 116/374 comparisons (31.0%) graph-derived | Every pooled graph-derived \|g\| > 5 source-checked |
| Dual digitization | Separate digitizer-level records not archived | Retrospective agreement statistic not calculable |
| Kang 2013 (90) | Eligible single-botanical arm separable; strict effect-size file checked | No Kang outcome and no combination arm pooled |

**Supplementary Table S7. Adapted GRADE certainty appraisal for preclinical animal evidence**

| Outcome | Risk of bias | Inconsistency | Indirectness | Imprecision | Publication bias | Overall certainty | Rationale |
| --- | --- | --- | --- | --- | --- | --- | --- |
| Femoral BMD | Serious | Serious | Serious | Serious | Serious concern | Very low | Most SYRCLE domains unclear; I² = 73.8% and PI crossed the null; heterogeneous preparations/models; dependence and Qi 2019 sensitivity; nine studies precluded reliable asymmetry testing. |
| BV/TV | Serious | Serious | Serious | Serious | Serious concern | Very low | Most SYRCLE domains unclear; I² = 79.5% and PI crossed the null; animal surrogate outcome and heterogeneous interventions; seven studies and a wide interval; selective dissemination cannot be excluded. |
| Tb.Th | Serious | Serious | Serious | Serious | Serious concern | Very low | Most SYRCLE domains unclear; I² = 79.2% and PI crossed the null; animal surrogate outcome and study-confounded preparation strata; publication bias remains possible. |
| Maximum load | Serious | Serious | Serious | Serious | Serious concern | Very low | Most SYRCLE domains unclear; I² = 63.0% and PI crossed the null; ex vivo surrogate outcome; only four studies with a wide interval; publication bias cannot be assessed reliably. |

Each outcome started at high certainty as experimental evidence and was downgraded across the five displayed domains; no upgrading factors were applied. Indirectness includes animal-to-human translatability. The appraisal was post hoc and follows Hooijmans et al. (2018).

**Supplementary Figure S2. Femoral BMD sensitivity and preparation-stratified analyses**

Panel A summarizes the primary model, Qi 2019 (100) exclusion, one-comparison-per-study analysis, and three-level cluster-robust model. The post hoc lower-risk rule identified only Li 2014 (93), so no pooled lower-risk sensitivity estimate was calculated. Panel B displays the three preparation strata with their 95% CIs; the overall primary DL estimate is repeated as a visual reference, and the interaction p value is shown in the figure. In both panels, the dashed red line denotes the primary DL estimate and the solid red line below the summary diamond denotes the 95% prediction interval. Sparse strata are exploratory and do not support comparative efficacy claims.
